# Association between the *MIF-173G/C* Polymorphism and Serum MIF levels with Pulmonary Tuberculosis: A Meta-analysis

**DOI:** 10.1038/s41598-017-00299-1

**Published:** 2017-03-22

**Authors:** Xiang Tong, Zhipeng Yan, Qilong Zhou, Sitong Liu, Jing Han, Yao Ma, Xue Yang, Hong Fan

**Affiliations:** 1 Department of Respiratory Medicine and Critical Care Medicine, West China Hospital/West China School of Medicine, Sichuan University, Chengdu, 610041 China; 2 Innovative Drug Research Centre, Chongqing University, Chongqing, 401331 China; 3 Department of Respiratory Medicine and Critical Care Medicine, Institute of Respiratory Disease, Guizhou Provincial People’s Hospital, Guiyang, 550000 China; 4 Department of Internal Medicine, No. 4 West China Teaching Hospital, Sichuan University, Chengdu, 610041 China

## Abstract

Many studies have indicated that *Macrophage migration inhibitory factor (MIF)-173G/C* gene polymorphisms are associated with susceptibility to pulmonary tuberculosis (PTB). Additionally, some studies have suggested that there are higher levels of serum MIF in patients with PTB than the controls. However, the results of these studies were underpowered. The current study aimed to precisely evaluate the association between the *MIF-173G/C* polymorphism and serum MIF concentrations with PTB. Therefore, a systematic literature search was preformed to identify studies involving the indicated association. Eleven articles (1316 cases and 1272 controls) were included in the study. The results indicated that the *MIF*-*173G/C* polymorphism was significantly associated with PTB susceptibility, especially in Asians. Interestingly, the results further detected that circulating MIF levels were significantly higher in patients with PTB than in healthy controls, but this was only the case among Asians. Moreover, the statistical significance was also similar to that of the high quality group. The present study indicated that the *MIF*-*173G/C* polymorphism may contribute to the development of PTB. Furthermore, significantly higher serum MIF levels were observed in PTB patients than in controls, which further indicated that the MIF may play an important role in PTB progression, particularly in Asians.

## Introduction

Tuberculosis (TB) is a serious global health problem that is mainly caused by the bacillus *Mycobacterium tuberculosis* (*Mtb*). According to a recent World Health Organization (WHO) report, there were an estimated 9.6 million new TB cases and 1.5 million TB deaths globally in 2014^1^. Currently, South-East Asia and the Western Pacific Regions account for 58% of the world’s TB cases^1^. India, China and Indonesia account for 23%, 10% and 10% of the total global cases, respectively^1^. Although approximately one-third of the world’s population is infected with *Mtb*, only approximately 10–15% of those infected have a risk of developing active disease at some later stage in life^2^. It is well known that a series of factors contribute to the risk of progression to infection and disease, which mainly include malnutrition, smoking, diabetes, alcohol use, human immunodeficiency virus (HIV) infection, socioeconomic status and environmental pollution, among others^3, 4^. Additionally, many studies have indicated that host genetic factors are also important determinants^5^.

Macrophage migration inhibitory factor (MIF), a 12.5-kDa protein, has been widely studied. Fifty years ago, MIF was first identified as a soluble factor that is produced by activated T lymphocytes that inhibit the random migration of macrophages *in vitro*, contributing to delayed hypersensitivity reactions^6^. During subsequent decades, many studies have confirmed MIF expression in a variety of cells and tissues (such as the pituitary, macrophages, dendritic cells, and neutrophils), except activated T cells^7^. In addition, MIF has been considered to have a variety of biologic functions, including macrophage activation, tumoricidal activities, glucocorticoid negativeregulation, pro-inflammatory activity and catalytic activity^8–10^. More recently, studies have found the MIF may played a vital role in the pathogenesis of infectious diseases, such as sepsis and Gram-negative bacterial infection^11^. Furthermore, significantly higher MIF concentrations were observed in patients with pulmonary tuberculosis (PTB) than in the healthy population^12^. *In vitro*, MIF has also been found to play an important role in restraining virulent *Mtb* growth^13^.

The human *MIF* gene is located on chromosome 22q11.2. One polymorphism (−173G/C, rs755622) in the *MIF* gene promoter with potential functional relevance has been identified^14^. A previous study indicated that individual subjects carrying a *MIF*-*173C* allele had significantly higher MIF production in the blood^15^. Several studies have demonstrated that the *MIF*-*173G/C* genetic variation may be associated with autoimmune diseases and cancer susceptibility^16, 17^. Moreover, the *MIF*-*173G/C* gene polymorphism was found to increase the risk of PTB^18, 19^. However, small sample sizes of some studies possibly lacked sufficient power to assess the true value. Therefore, to the best of our knowledge, a question of whether the *MIF*-*173G/C* gene polymorphism and serum MIF levels are associated with PTB risk has not been systematically explored.

A meta-analysis is the statistical analysis of a large collection of results from multiple original studies to synthesize their findings. It has the advantage of encompassing large subject numbers, increasing the ability to detect small but important effects. In the current study, we performed a systematic review and meta-analysis to accurately investigate the impact of the *MIF*-*173G/C* gene polymorphism and serum MIF levels on PTB susceptibility.

## Results

### Study characteristics

**Figure 1:**
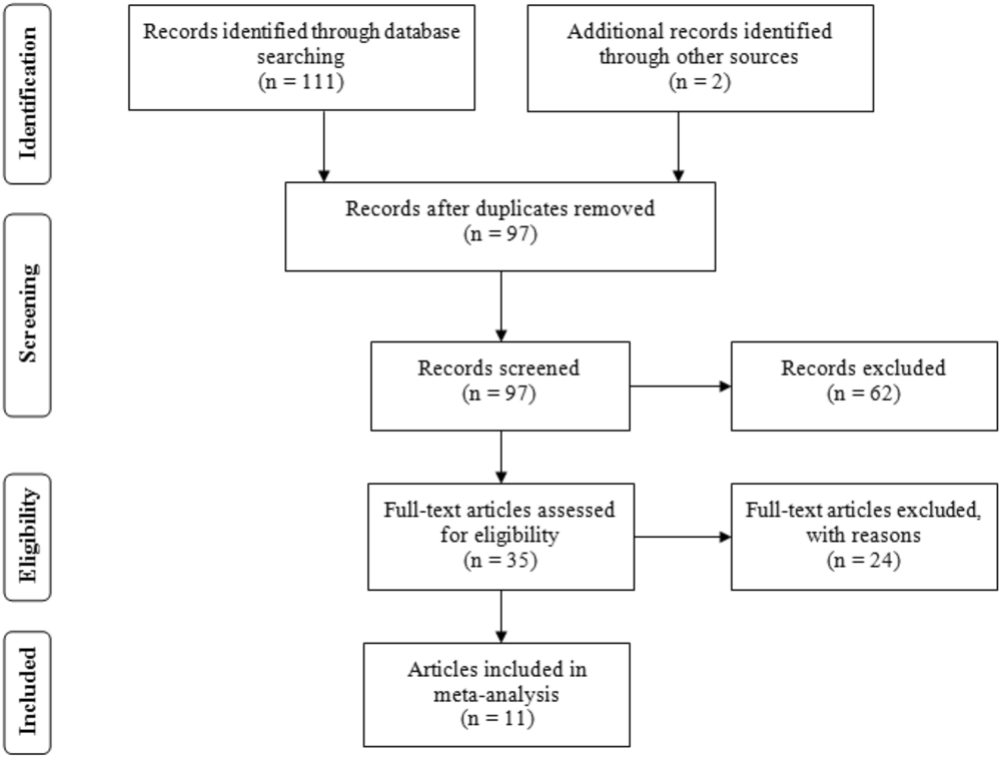
The flow diagram of the included and excluded studies.

Depending on the search strategy, we identified 113 articles when we initially searched the PubMed, Embase, CNKI, and Wanfang databases as well commercial Internet search engines. As shown in Fig. 1, sixteen studies were excluded because they were duplicate studies. Sixty-two articles were excluded based on their titles and abstracts. After full-view screening, fourteen articles were excluded because they were not relevant to PTB risk in relation to the *MIF*-*173G/C* gene polymorphism and/or serum MIF concentrations. Two articles were excluded because they were reviews. Five articles were excluded because they were potential repeat studies. Two studies were excluded because they were animal experiments. Another single article was not included in the meta-analysis because it was not designed as a case-control study. Finally, 11 eligible articles^12, 18–27^ were included in the current meta-analysis. Nine of the 11 included articles were in English^12, 18–24, 27^ and two were in Chinese^25, 26^. Among the studies, seven articles^12, 19, 23–27^ were conducted in Asian populations, three studies^18, 20, 21^ were conducted in Caucasians and one study^22^ was conducted in Africans. Kibiki *et al*. recruited PTB-HIV patients as participants to participate in this clinical study^22^. The included subjects in the four studies were free of HIV^12, 18, 20, 26^, whereas the comorbidity (HIV) was not addressed in another six studies^19, 21, 23–25, 27^. Additionally, three articles were of moderate quality (score = 6)^24, 26, 27^, and the other included studies were of high quality according to the Newcastle-Ottawa Scale quality score evaluation (see Methods)^12, 18–23, 25^. The characteristics of the collected studies are listed in Tables 1 and 2.

**Table 1:** Characteristics of the studies involving an association between the *MIF-173G/C* polymorphism and PTB risk included in the meta-analysis.

Author	Year	Coutry	Ethnicity	P^1^/C^2^	Age	HIV	Case			Control			Method	Score
							CC	CG	GG	CC	CG	GG		
Gomez LM^20^	2007	Colombia	Caucasian	230/235	40 ± 16/43 ± 16	No	15	95	120	16	86	133	PCR-Sequencing	7
Hashemi M^21^	2015	Iran	Caucasian	161/142	50.6 ± 20.5/47.3 ± 15.4	NR	9	53	99	5	32	105	PCR-RFLP^4^	8
Kuai SG^23^	2015	China	Asian	47/50	NR^3^	NR	25		22	16		34	PCR-RFLP	7
Li Y^19^	2012	China	Asian	215/245	43.8 ± 5.76/40.2 ± 4.90	NR	32	74	109	16	61	168	PCR-RFLP	7
Liu AH^27^	2015	China	Asian	200/100	40.56 ± 17.39/38.65 ± 9.15	NR	4	116	80	6	37	57	PCR-RFLP	6
Sadki K^18^	2010	Morocco	Caucasian	123/154	33.30 ± 13.09/32.89 ± 11.20	No	21	45	57	9	63	82	RT-PCR^5^	7

^1^patient; ^2^control; ^3^not report; ^4^polymerase chain reaction-restriction fragment length polymorphism; ^5^real time-polymerase chain reaction.

**Table 2:** Characteristics of the studies involving the association between the serum MIF levels and PTB included in the meta-analysis.

Author	Year	Country	Ethnicity	P^1^/C^2^	Age	HIV	Case			Control			Unit	Method	Score
							Mean	SD^4^	N	Mean	SD	N			
Dai XL^25^	2011	China	Asian	41/41	21-42/19-44	NR	43.24	29.29	41	20.86	11.99	41	ng/ml	ELISA^4^	7
Kibiki GS^22^	2007	Tanzania	African	27/46	39.2 ± 8.9/39.3 ± 10.6	Yes	130.50	71.11	27	136.4	104.3	46	ng/ml	ELISA	7
Kibiki GS^22^	2007	Tanzania	African	25/25	33.2 ± 13.8/38.7 ± 10.9	No	47.60	87.48	25	39.30	23.93	25	ng/ml	ELISA	7
Kuai SG^23^	2015	China	Asian	47/50	NR^3^	NR	17.19	6.64	47	9.36	5.29	50	ng/ml	ELISA	7
Li Y^24^	2012	China	Asian	151/149	NR	NR	705.2	67.98	151	355.3	57.29	149	pg/ml	ELISA	6
Liu AH^27^	2014	China	Asian	200/100	40.3 ± 17.9/28.7 ± 9.2	NR	9.67	7.24	200	4.57	1.89	100	ng/ml	ELISA	6
Yamada G^12^	2002	Japan	Asian	34/30	54 ± 20/47 ± 10	No	19.84	11.27	34	4.38	1.34	30	ng/ml	ELISA	7
Zhao GX^26^	2012	China	Asian	62/55	37.5 ± 12.3/35.4 ± 10.5	No	465.46	51.15	65	273.82	37.48	55	pg/ml	ELISA	6

^1^patient; ^2^control; ^3^not reported; and ^4^enzyme-linked immunosorbent assay.

### Association between PTB and the *MIF*-*173G/C* gene polymorphism

**Figure 2:**
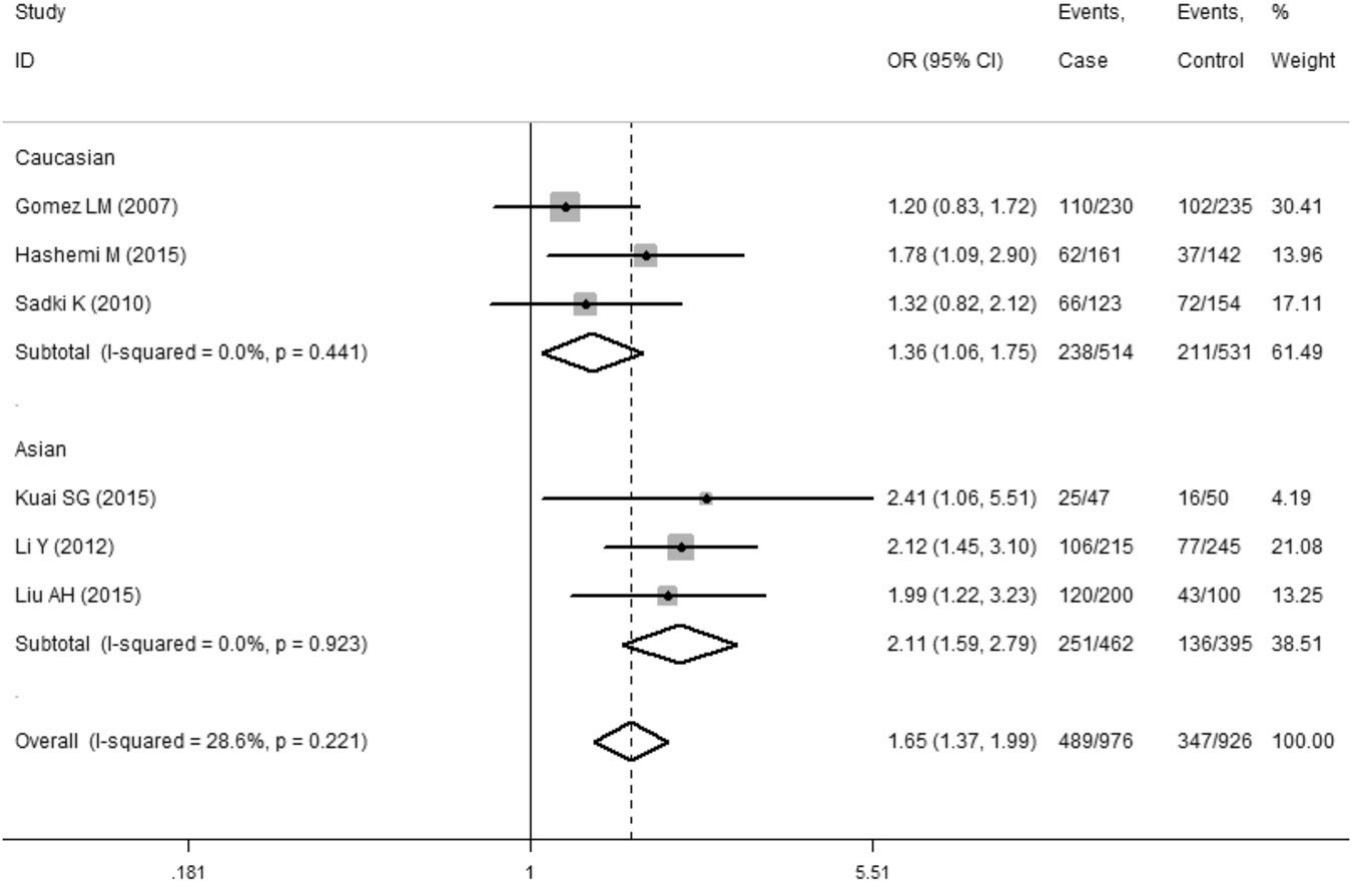
Meta-analysis results of the association between the PTB risk and *MIF-173G/C* gene polymorphism (CC + CG vs. GG).

**Figure 3:**
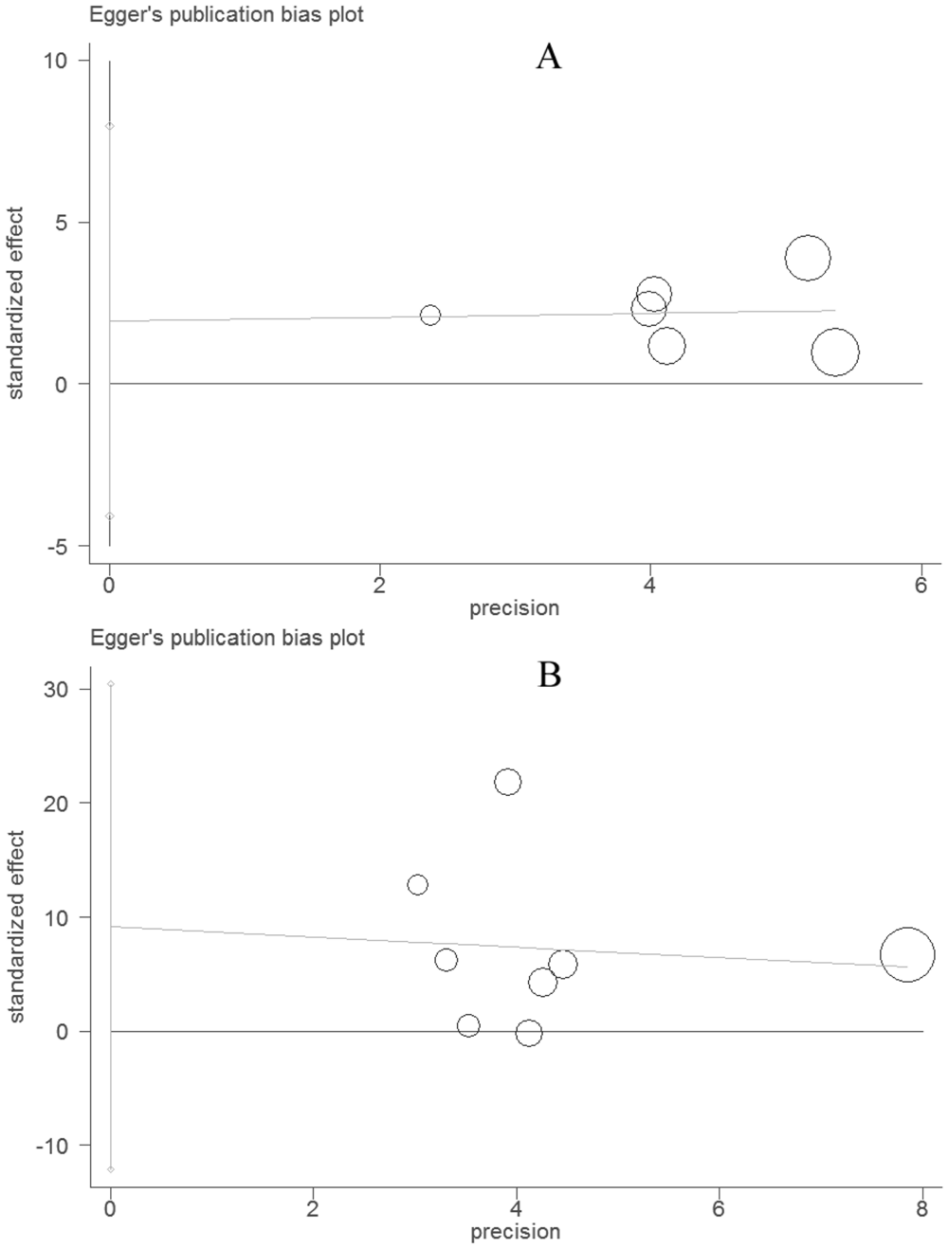
Funnel plot for evaluating publication bias on the association between the PTB risk and MIF-173G/C gene polymorphism (**A**) as well as on the association between the serum MIF levels and PTB (**B**). Each circle represents a separate study for the indicated association.

In total, six studies (976 cases and 926 controls) reported an association between the *MIF*-*173G/C* gene polymorphism and PTB susceptibility. In the overall meta-analysis, the fixed-effect model was used in the dominant (CC + CG vs. GG), co-dominant (CG vs. GG), and allele (C vs. G) genetic models, while the random-effect model was applied in the co-dominant (CC vs. GG) and recessive (CC vs. CG + GG) genetic models. As summarized in Table 3, the results indicated that there was evidence for significant associations between PTB and the *MIF*-*173G/C* gene polymorphism in the dominant (CC + CG vs. GG, OR = 1.65, 95%CI = 1.37–1.99, P < 0.001), co-dominant (CG vs. GG, OR = 1.54, 95%CI = 1.26–1.88, P < 0.001), and allele (C vs. G, OR = 1.49, 95%CI = 1.28–1.74, P < 0.001) genetic models. In a stratified analysis by specific ethnicity, there were significant associations between the *MIF*-*173G/C* gene polymorphism and PTB risk for the dominant, co-dominant (CG vs. GG), and allele genetic models in Asians, but that was only the case for the dominant and allele genetic models (Fig. 2 and Table 3) in Caucasians. When a subgroup analysis was performed by the study quality specific effect, there were significant associations between the *MIF*-*173G/C* gene polymorphism and PTB risk in the high quality group (CC + CG vs. GG, OR = 1.60 95%CI = 1.31–1.95, P < 0.001) (Table 3). No publication bias was evaluated by either the Begg’s (P = 0.452) or Egger’s test (P = 0.419, Fig. 3A).

**Table 3:** Summary of the overall and subgroup analysis results from different comparative genetic models.

Genetic model	Overall		Asian		Caucasian		High quality	
	OR^1^ (95%CI^2^)	P	OR (95%CI)	P	OR (95%CI)	P	OR (95%CI)	P
CC + CG vs. GG	1.65 (1.37–1.99)	<0.001	2.11 (1.59–2.79)	<0.001	1.36 (1.06–1.75)	0.015	1.60 (1.31–1.95)	<0.001
CC vs. CG + GG	1.46 (0.73–2.93)	0.286	0.97 (0.13–7.23)	0.975	1.70 (0.77–3.77)	0.188	1.91 (1.09–3.36)	0.024
CC vs. GG	1.73 (0.90–3.32)	0.098	1.32 (0.21–8.21)	0.763	1.83 (0.87–3.85)	0.110	2.18 (1.48–3.22)	<0.001
CG vs. GG	1.54 (1.26–1.88)	<0.001	2.01 (1.46–2.77)	<0.001	1.28 (0.99–1.67)	0.061	1.43 (1.14–1.78)	0.002
C vs. G	1.49 (1.28–1.74)	<0.001	1.70 (1.18–2.46)	0.004	1.34 (1.10–1.64)	0.004	1.53 (1.16–2.01)	0.002

^1^odds ratio and ^2^confidence interval.

### Association between PTB and the serum MIF levels

**Figure 4:**
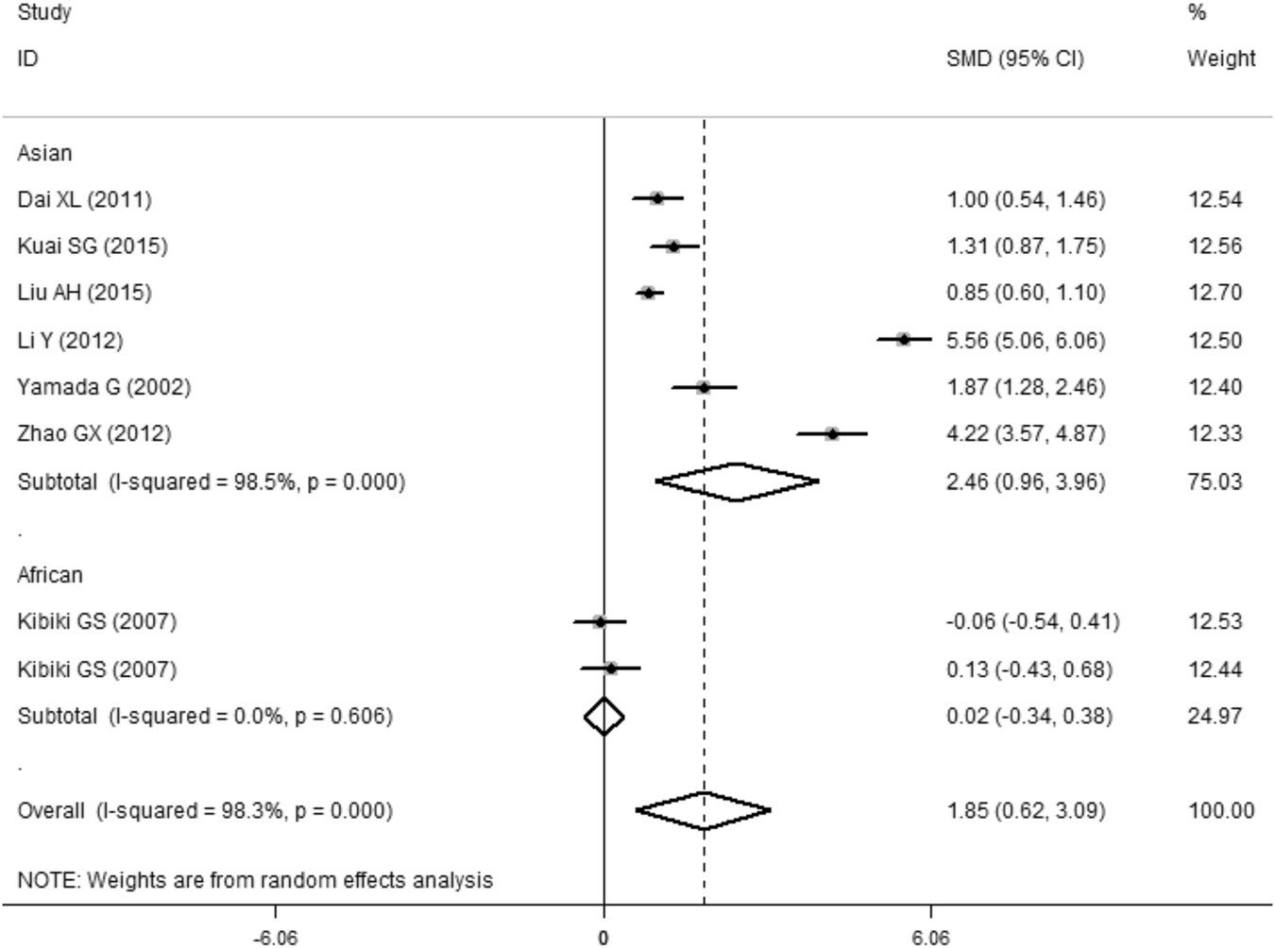
Meta-analysis results of the association between the serum MIF levels and PTB.

**Figure 5:**
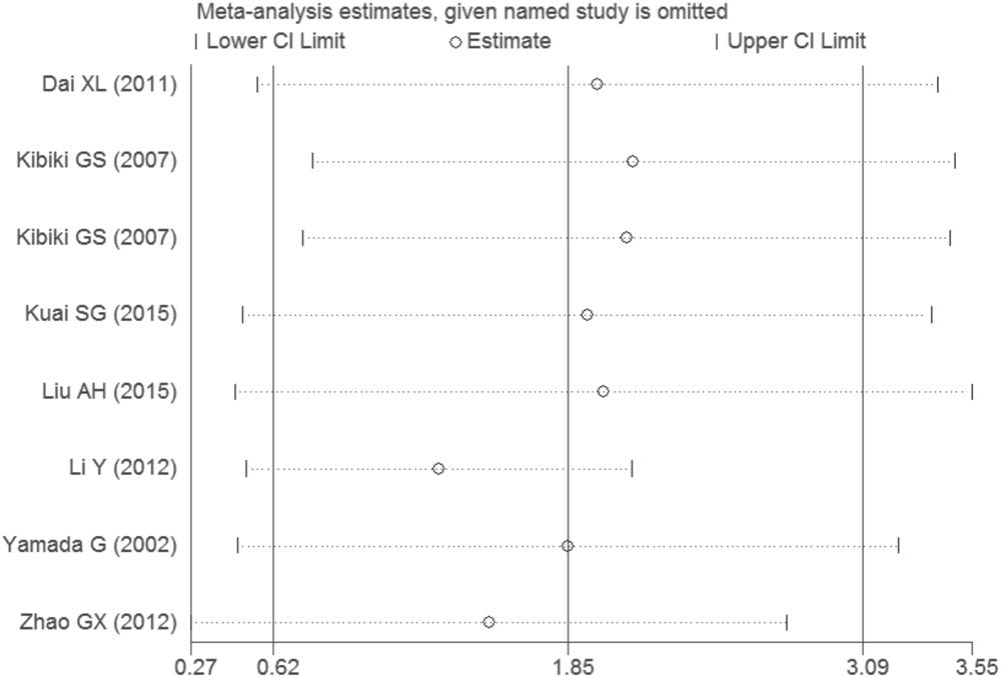
Sensitivity analysis result of the association between the serum MIF levels and PTB.

All 8 eligible case-control studies (587 cases and 496 controls) from seven articles showed an association between PTB and the serum MIF concentrations. Overall, the meta-analysis results indicated that the serum MIF levels in patients with PTB were significantly higher than those in healthy controls (SMD = 1.85, 95%CI = 0.62–3.09, P = 0.003) (Table 4 and Fig. 4). However, a non-ignorable heterogeneity among studies was observed (I^2^ = 98.3%). Although we conducted the overall meta-analysis by the random-effect model, the results are required for further analysis. As a result, we performed subgroup analyses by ethnicity-specific effects. The serum MIF levels in Asian patients with PTB were significantly higher than those in healthy controls (SMD = 2.46, 95%CI = 0.96–3.96, P = 0.001), but not in Africans (SMD = 0.02, 95%CI = −0.34–0.38, P = 0.921). The statistical significance was similar to that of the high quality group when we performed subgroup analysis according to the study quality (SMD = 0.84, 95%CI = 0.17–1.52, P = 0.014). Moreover, we further executed a sensitivity analysis by sequentially excluding studies from the meta-analysis to investigate the influence of each study on the pooled results. The results of the sensitivity analysis revealed that the pooled OR was not materially altered (Fig. 5). There was no any publication bias as calculated by the Begg’s (P = 0.266) and Egger’s tests (P = 0.332, Fig. 3B).

**Table 4:** The pooled results of the serum MIF levels in PTB patients compared with healthy controls.

	Samples (patients/controls)	Number of studies	SMD^1^	95%CI^2^	P	I^2^	Model
Overall	587/496	8	1.85	0.62–3.09	0.003	98.3	Random
Asian	535/425	6	2.46	0.96–3.96	0.001	98.5	Random
African	52/71	2	0.02	−0.34–0.38	0.921	0	Fixed
High quality	174/192	5	0.84	0.17–1.52	0.014	89.1	Random

^1^standardized mean difference and ^2^confidence interval.

## Discussion

In the current meta-analysis, six case-control studies involving the relationship between the *MIF*-*173G/C* gene polymorphism and PTB risk were included. The overall results indicated that the individuals with the variant C allele in the *MIF* gene had increased PTB susceptibility. In addition, subgroup analyses found that the *MIF*-*173G/C* gene polymorphism was associated with PTB susceptibility in Asians, and the study quality did not significantly impact the results.

Interestingly, there were eight primary case-control studies that compared the blood MIF concentrations in patients with PTB and in healthy controls. The overall results of this meta-analysis indicated that patients with PTB had higher serum MIF levels than controls. However, we found a very significant heterogeneity (I^2^ = 98.3%) between the studies in the meta-analysis, which may be attributed to many factors, as follows: (1) different demographic and genetic characteristics of the Caucasian, Asian and African populations; (2) different quality of the included studies; (3) different co-morbidity in each study; and (4) different diagnosis and inclusion criteria for each primary study. Although the heterogeneity can be taken into account with the random-effect model, it would increase the probability of Type I error. To further identify the reasons for heterogeneity, we first carried out a sensitivity analysis by sequentially excluding each study. Statistically similar results were obtained, suggesting the stability of the meta-analysis. Second, we conducted subgroup analysis by ethnicity and study quality.

On the topic of the association between PTB and the serum MIF levels, the included studies were mainly conducted in Asians (six case-control studies). Subgroup analysis by ethnicity found that Asian patients with PTB had higher serum MIF levels than the healthy population. However, the blood MIF concentrations did not increase in Africans with PTB compared to the control subjects. We do not need to note that the result may have insufficient power to reveal a reliable value because only one article in an African population was included. Additionally, we also observed that patients with PTB still had higher serum MIF concentrations than in the healthy population when we combined the high quality studies. The heterogeneity was still detected after stratified analysis. Although the heterogeneity was still detected after stratified analyses by ethnicity and study quality, subgroup analyses may provide more precise results than the overall analysis.

MIF is produced by a variety of cells and tissues, and it is rapidly released after exposure to microorganisms (toxins and cell wall components) and pro-inflammatory mediators as well as in response to stress^7, 9, 28^. Additionally, MIF had been shown to play a crucial role in the trafficking and regulation of innate and adaptive immunity^9, 29, 30^. In previous studies, we found that the *MIF* gene variant may be associated with susceptibility to cancer and renal disease for which cytokines and immuno-regulation play an important role in pathogenesis^17, 31^.

Furthermore, many earlier studies found that macrophages and activated T lymphocytes release MIF, which promotes a range of pro-inflammatory cytokines, such as TNF-α after *Mtb* infection^32, 33^. More recently, a study suggested that the MIF knock-out mice succumbed more quickly with a higher *Mtb* burden, decreased innate immunity cytokine expression (such as TNF-α and interleukin-12), and impaired *Mtb* killing^34^. Another study demonstrated that MIF produced by infected human macrophages inhibited the growth of virulent *Mtb* ^13^. In the clinical study conducted by Yamada and co-workers, the authors found that the mean levels of blood MIF were significantly higher in those with PTB than in healthy participants. Additionally, they suggested that the circulating MIF values significantly correlated with serum interferon-γ, which is one of the most important cytokines contributing to protective immunity against *Mtb* ^12^. Similar results were found by Li and co-colleagues in a Chinese population^24^. Fortunately, our meta-analysis results were consistent with the previous consequences, suggesting that MIF may played a crucial role in immune responses to individual infection with *Mtb*. Additionally, Donn *et al*. reported that the serum levels of MIF were significantly higher in individual carrying a *MIF*-173C allele^15^. Interestingly, we also found that the *MIF-173G/C* gene polymorphism may be a risk factor contributing to PTB susceptibility in the current meta-analysis. Although the mechanism by which serum MIF and the *MIF*-173G/C gene polymorphism are involved in PTB development in humans remains completely unclear, the results of the present and previous studies may help us to identify new molecular markers for TB diagnosis and targets for treatment.

There are several limitations to this meta-analysis. First, even if no publication bias was observed by Egger’s test, only published studies were identified in a few databases. As a result, there may be other biases in the present study. Second, we are unable to further perform subgroup analysis to investigate the other factors (gene-environment interaction, gender and age, etc.) that may affect our results because individual level data are not available. Third, because most studies have not reported the comorbidity (HIV), we did not perform a subgroup analysis on the comorbidity-specific effect. Despite these limitations, we minimized the likelihood of bias through the entire process by establishing a detailed protocol and performing study identification, data selection, and statistical analysis as well as controlling for publication bias. In any case, the reliability of the results is assured.

In conclusion, the current study suggested that the *MIF*-*173G/C* gene polymorphism may be a risk factor contributing to PTB susceptibility. Moreover, significantly higher serum MIF levels were detected in PTB patients than in controls, which further indicates MIF possibly played an important role in developing PTB, particularly in Asians. We strongly recommend that researchers design more rigorously and uniformly case-control or cohort studies to confirm the results in the future.

## Methods

### Literature search

We performed a systematic literature search in the PubMed, Embase, and Wanfang databases and China National Knowledge Internet (CNKI) to identify studies involving the association between the *MIF*-*173G/C* gene polymorphism and/or blood MIF concentrations with PTB susceptibility up to October 12, 2016. The key terms in the search were as follows: (“tuberculosis” OR “pulmonary tuberculosis” or “TB” or “PTB”) and (“macrophage migration inhibitory factor” or “MIF”). The language was restricted to English and Chinese. Additionally, we conducted a Web-based search of all types of commercial Internet search engines (such as Google and Baidu) using the same technique. Furthermore, the reference lists of the obtained articles were also reviewed.

### Study selection

The inclusion criteria were as follows: (1) the study should be designed as a case–control study; (2) the study should evaluate the association between the *MIF*-*173G/C* gene polymorphism and/or serum MIF levels and PTB risk; (3) the available data for calculating the odds ratio (OR) and standardized mean difference (SMD) with a 95% confidence interval (CI) should be provided in the primary study; and (4) the study subjects should be human. The exclusion criteria were as follows: (1) lack of a control cohort; (2) review and overlapping study; and (3) the study does not show the available data and other essential information.

### Quality score evaluation

The qualities of the included studies were assessed by the Newcastle-Ottawa Scale (case-control study), a scale that is used to estimate quality based on three aspects, including selection, comparability and exposure in the primary study. The total score ranged from 0 to 9 (0–3, 4–6, and 7–9 were considered low, moderate, and high quality, respectively).

### Date extraction

Two independent authors (Xiang Tong and Zhipeng Yan) collected the detailed information and data from each study by a pre-designed data extraction Excel form. If there was a disagreement, the third author (Qilong Zhou) settled the disagreement. The information and data were extracted as follows: first author, publication year, country, ethnicity, sample size, age, genotype distribution, serum MIF levels, comorbidity (HIV) and test method.

### Statistical methods

In the present study, the OR and 95% CI were used to investigate the effect strength of the association between the *MIF*-*173G/C* gene polymorphism and PTB susceptibility, while the SMD was applied to compare the serum MIF levels in the patients with PTB and healthy controls. We calculated the heterogeneity using the χ^2^ based Q-test and I-squared (I^2^) statistics test. The pooled effect size (OR and SMD) was assessed by the random-effect model if heterogeneity was considered statistically significant (I^2^ value more than 50% and P value less than 0.10). If not, the fixed-effect model was used. To evaluate the ethnicity and study quality specific effects, we also performed subgroup analysis by different specific effects.

In addition, publication bias was evaluated by several methods. The Begg’s and Egger’s tests were used to assess publication bias^35, 36^. Visual inspection of asymmetry in funnel plots was also performed to further investigate the publication bias. All data analyses were performed by STATA 12.0 software^37^.
